# Supplementary material for: High density genomic surveillance and risk profiling of clinical Listeria monocytogenes subtypes in Germany
Source: Genome Med. 2024 Oct 7;16:115. doi: 10.1186/s13073-024-01389-2 (PMC11457394; doi:10.1186/s13073-024-01389-2)
Supplement: Supplementary file 1 — Supplementary Material 1: Fig. S1. Comparison of the population structures of clinical L. monocytogenes isolates from in- and outside Germany. Fig. S2. Clinical disease manifestation as reported during case notification for different serogroups. Fig. S3. Duration and geographical spread of listeriosis clusters. Fig. S4. Absence of an Epsilon1b prophage in the L. monocytogenes Epsilon1a outbreak clone. Table S1. Clinical L. monocytogenes isolates included in this study. Table S2. Genomic features of representative isolates from selected German outbreak clusters. Table S3. Subpopulations of clinical L. monocytogenes isolates with inactivating mutations in selected genes. [file 13073_2024_1389_MOESM1_ESM.zip › halbedel et al. supplementary information.pdf]

Supplementary Information

to

High density genomic surveillance and risk profiling of clinical *Listeria monocytogenes* subtypes in Germany

by

Sven Halbedel, Sabrina Wamp, Raskit Lachmann, Alexandra Holzer, Ariane Pietzka, Werner Ruppitsch, Hendrik Wilking, Antje Flieger

Content

Fig S1: Comparison of the population structures of clinical *L. monocytogenes* isolates from in- and outside Germany.
Fig S2: Clinical disease manifestation as reported during case notification for different serogroups.
Fig S3: Duration and geographical spread of listeriosis clusters.
Fig S4: Absence of an Epsilon1b prophage in the *L. monocytogenes* Epsilon1a outbreak clone.

Table S1: Clinical *L. monocytogenes* isolates included in this study (**please see separate excel file**).
Table S2: Genomic features of representative isolates from selected German outbreak clusters.
Table S3: Subpopulations of clinical *L. monocytogenes* isolates with inactivating mutations in selected genes.

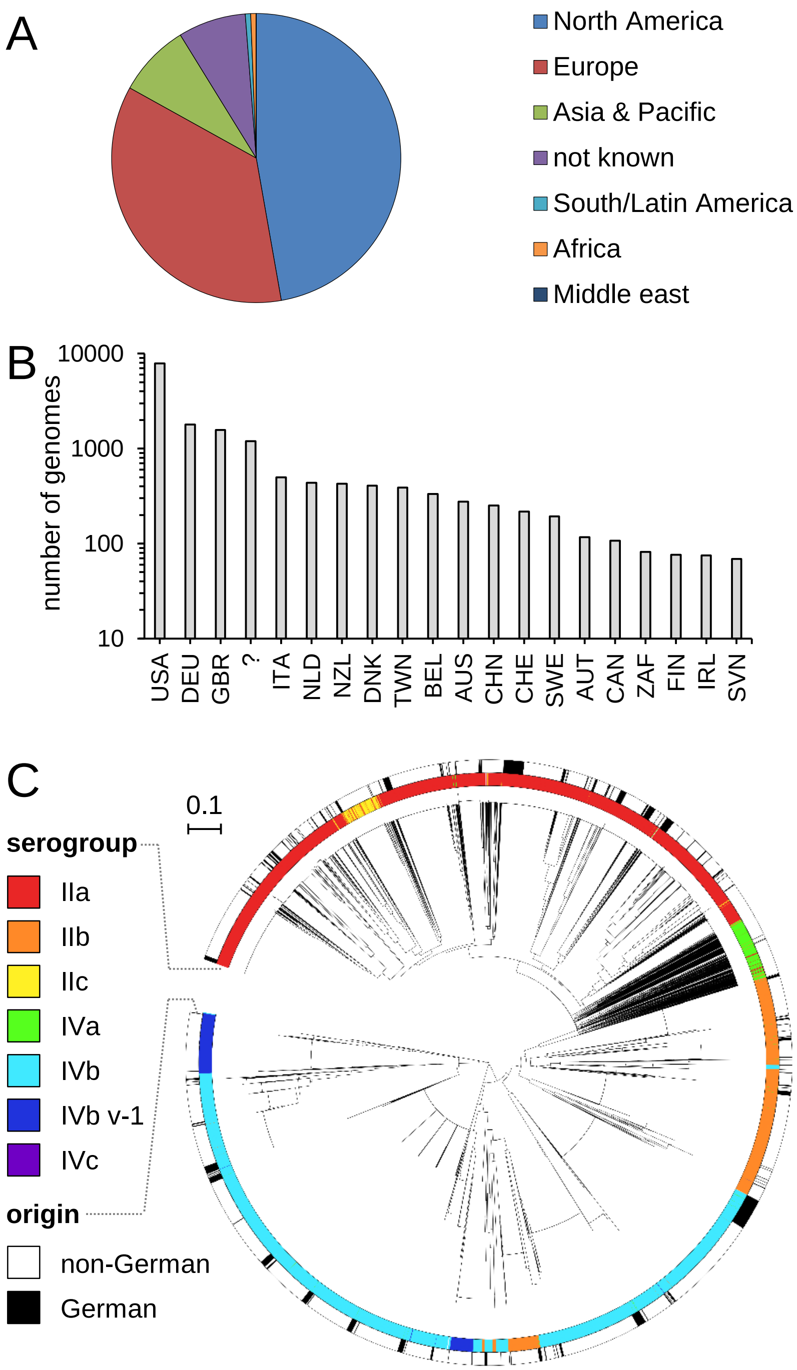

Fig S1: Comparison of the population structures of clinical *L. monocytogenes* isolates from in- and outside Germany.
(A) Representation of the different world regions in the dataset used for comparison. 15,155 non-German NCBI isolate genomes and 1,802 isolate genomes from Germany are included.
(B) Diagram illustrating the countries of isolation in the same dataset. Countries with the most isolates are shown, ? – country of isolation not known.
(C) Unrooted neighbor joining tree calculated on seven locus MLST data showing the phylogenetic structure of the 15,155 non-German and the 1,802 German clinical isolates. Tree tips are colored according to molecular PCR serogroups (inner ring) and to their geographic origin (outer ring). The scale bar indicates the number of allelic substitutions per locus.

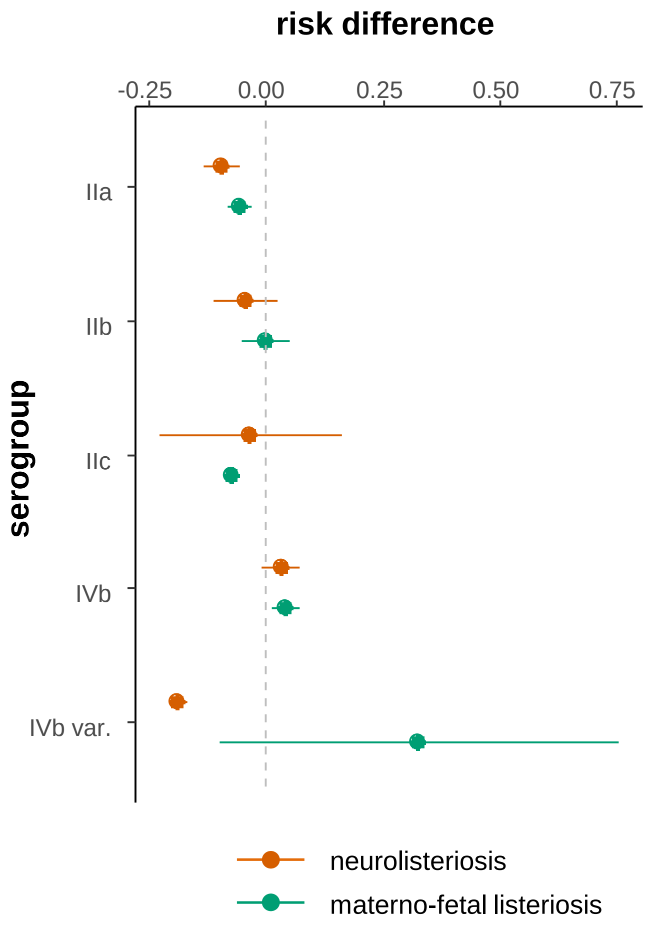

Fig S2: Clinical disease manifestation as reported during case notification for different serogroups.
Risks for NL and MFL manifestations are expressed as risk differences together with 95% confidence intervals for the individual serogroups. Data were calculated based on 1,323 isolate/notification case pairs, for which information on the disease manifestation were available.

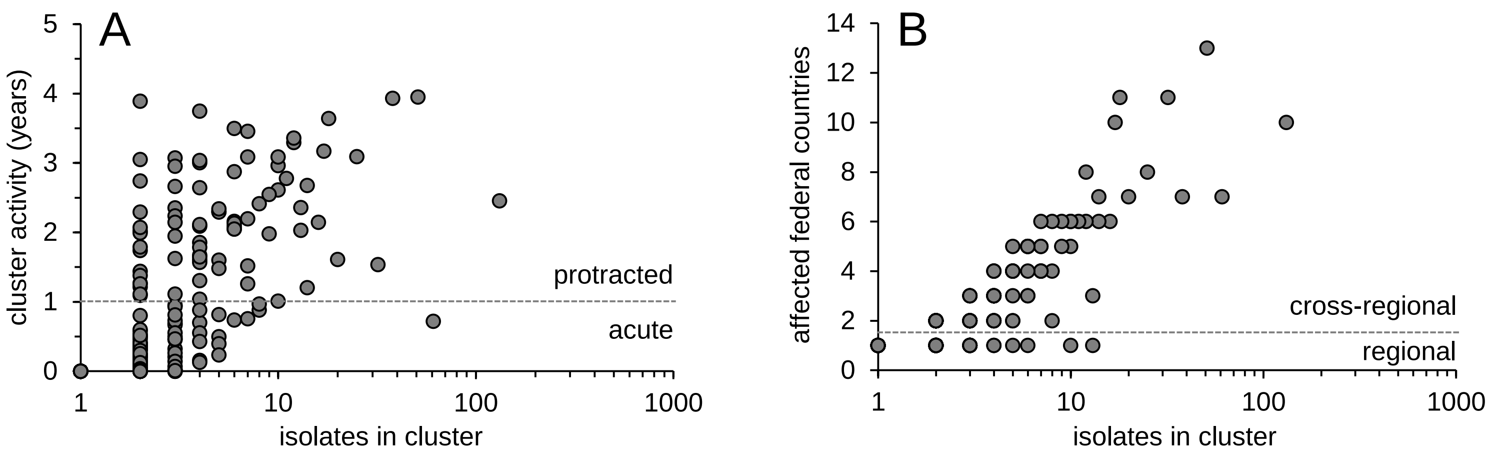

Fig S3: Duration and geographical spread of listeriosis clusters.

(A) Diagram showing the duration of all listeriosis clusters within the four-year study period plotted against the number of isolates.

(B) Diagram illustrating the geographical distribution by plotting the number of involved federal countries in Germany against cluster size.

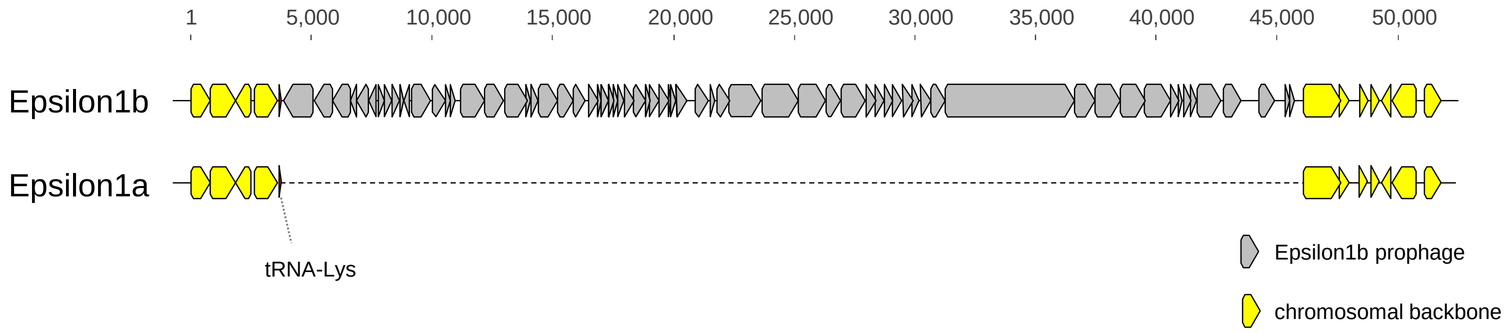

Fig S4: Absence of an Epsilon1b prophage in the *L. monocytogenes* Epsilon1a outbreak clone.
Comparison of the tRNA-Lys chromosomal regions of strains 18-04540 (Epsilon1a, accession number: CP063383) and 11-04869 (Epsilon1b, accession number: CP110922).

Table S2: Genomic features of representative isolates from selected German outbreak clusters.

	**Alpha10**	**Epsilon1b**	**Theta3a**	**Kappa8**	**Eta7**
strain	21-03201	11-04869	16-02236	19-07394	19-02390
source type	clinical isolate	clinical isolate	clinical isolate	clinical isolate	clinical isolate
isolation source	not reported	blood	CSF[1]	blood	nasal swap
year of isolation	2021	2011	2016	2019	2019
NCBI accession	CP111149	CP110922	CP111148	CP113891	CP111150
ENA accession	ERS14238016	ERS2103006	ERS14238017	ERS14291506	ERS14238018
phyl. lineage	I	I	I	I	I
molecular serogroup	IVb	IVb	IVb	IVb	IVb
ST/CC[1]	ST1/CC1	ST6/CC6	ST249/CC315	ST1/CC1	ST6/CC6
CT[2] Ruppitsch	CT6329	CT90	CT4449	CT4961	CT7504
CT[2] Pasteur	CT9528	CT443	CT12857	CT3989	CT7198
sequencing method	NextSeq/MinION	MiSeq/MinION	MiSeq/MinION	MiSeq/MinION	MiSeq/MinION
long read raw data	ERR10513163	ERR10513160	ERR10513161	ERR10556194	ERR10513162
short read raw data	ERR10481070	ERR2261336	ERR10481069	ERR10556193	ERR10481068
genome size	2,946,483 bp	3,034,959 bp	2,976,730 bp	2,947,414 bp	2,956,565 bp
G/C content	38.0%	38.0%	38.0%	38.0%	38.0%
CDS[3] (protein)	2,850	2,988	2,851	2,852	2,860
rRNA operons	6	6	6	6	6
tRNA genes	67	67	67	67	65
PAIs[3]	LIPI-1, LIPI-3	LIPI-1, LIPI-3	LIPI-1, LIPI-4	LIPI-1, LIPI-3	LIPI-1, LIPI-3
SSIs[3]	-	-	SSI-1	-	-
plasmids	-	pLMST6 (CP110923)	-	-	-
intact prophages[4]	1	3	1	1	1
insertion sites	*comK*	$tRNA^{Lys}$ (*lmot01*)<br>OQ358_06820<br>*rpsI*	$tRNA^{Arg}$ (*lmot17*)	$tRNA^{Lys}$ (*lmot01*)	*tsf*
PMSCs[3]	ORY88_00790 (*lmo0140*)<br>ORY88_10650 (*lmo2084*)	OQ358_01125 (*lmo0140*)<br>OQ358_02035 (*lmo0310*)<br>OQ358_02370 (*lmo0380*)<br>OQ358_02740 (*lmo0440*)	ORY89_02055 (*lmo0380*)<br>ORY89_07620 (*ispG*)<br>ORY89_12510 (*lmo2409*)	OZX46_01155 (*lmo0140*)<br>OZX46_03895 (*lmo0671*)<br>OZX46_11015 (*lmo2084*)<br>OZX46_14505 (*lmo2797*)	ORY90_00355 (*essC*)<br>ORY90_00795 (*lmo0140*)<br>ORY90_01705 (*lmo0310*)<br>ORY90_02040 (*lmo0380*)<br>ORY90_02410 (*lmo0440*)<br>ORY90_03140 (*lmo0595*)<br>ORY90_05765 (*lmo1123*)<br>ORY90_05820 (*lmo1134*)<br>ORY90_14515 (*lmo2781*)

[1] ST - sequence type (ST) and clonal complex (CC) according to seven locus MLST (1),
[2] CT - complex types according to Ruppitsch and Pasteur cgMLST schemes (2, 3),
[3] abbreviations: CSF - cerebrospinal fluid, CDS – coding sequences, PAI – pathogenicity associated island, PMSC – premature stop codon, SSI – stress survival islet,
[4] according to PHASTER (4).

Table S3: Subpopulations of clinical *L. monocytogenes* isolates with inactivating mutations in selected genes.

gene	name	function	mutation	affected subgroup[1]	expected phenotype	number of isolates[2]
lmo0105	*chiB*	chitinase	S345X	Iota6 (CT6520, ST4, IVb)	no chitin utilization (5)	2/2
				Iota7 (CT8189, ST4, IVb)	virulence defect in mice (6)	2/2
				Tau4 (CT6460, ST4, IVb)		5/5
lmo0409	*inlF*	internalin F	Q451X	Tau8 (CT9031, ST451, IIa)	host cell entry defect (7)	16/16
lmo0433	*inlA*	internalin A	E326X	My5 (CT6466, ST9, IIc)	host cell entry defect (8)	2/2
			Q492X	ST121 (IIa)		15/15
			N546fs	Gamma11 (CT1698, ST9, IIc)		2/2
				Rho3 (CT1690, ST9, IIc)		3/4
lmo1076	*aut*	Auto autolysin	K132X	Psi2a/b (CT1234, ST155, IIa)	host cell entry defect (9)	3/3
			S144X	Omikron11 (CT14992, ST155, IIa)		2/2
			W163X	Delta8 (CT4295, ST121, IIa)		4/4
			N189fs	Pi5 (CT6665, ST3, IIb)		5/5
			N339fs	Alpha5 (CT6583, ST3, IIb)		3/3
lmo1138	*clpP1*	accessory ClpP1 protease	L34X	ST16 (IIa)	protein turnover defect (10)	9/9
lmo1412	*flaR*	required for flagellin expression	W50X	ST3 (IIb)	motility defect (11)	25/29
			D67fs	ST29 (IIa)		9/11
				ST37 (IIa)		58/64
				ST427 (IIa)		7/9
				ST1344 (IIa)		2/2
			K75fs	ST18 (IIa)		13/13
				ST26 (IIa)		13/15
				ST200 (IIb)		5/5
				ST207 (IIa)		3/4
lmo1441	*ispG*	hydroxymethylbutenyl diphosphate synthase	R115X	ST249 (IVb)	virulence defect in mice (12)	24/24
lmo2467		chitin binding protein	S82X	My5 (CT6466, ST9, IIc)	virulence defect in mice (6)	2/2
			W224X	ST29 (IIa)		8/11
lmo2550	*csbB*	required for WTA decoration with GlcNAc	A81fs	Sigma5 (CT5715, ST504, IIa)	GlcNAc-less WTA (13)	12/12
			L104fs	Omega5 (CT1138, ST87, IIb)		3/11
lmo2769	*eslA*	ABC transporter, ATP-binding protein	I160fs	ST38 (IIa)	lysozyme sensitive (14)	3/3
			I229fs	ST427 (IIa)		9/9

[1] STs associated with reduced MFL and NL risks were marked by a double underscore, those associated with reduced NL or MFL risk with a single underscore.
[2] Only mutations, which were found at least twice per phylogroup, were included.

References

1. Ragon M, Wirth T, Hollandt F, Lavenir R, Lecuit M, Le Monnier A, Brisse S. 2008. A new perspective on *Listeria monocytogenes* evolution. PLoS Pathog 4:e1000146.
2. Ruppitsch W, Pietzka A, Prior K, Bletz S, Fernandez HL, Allerberger F, Harmsen D, Mellmann A. 2015. Defining and Evaluating a Core Genome Multilocus Sequence Typing Scheme for Whole-Genome Sequence-Based Typing of *Listeria monocytogenes*. J Clin Microbiol 53:2869-76.
3. Moura A, Tourdjman M, Leclercq A, Hamelin E, Laurent E, Fredriksen N, Van Cauteren D, Bracq-Dieye H, Thouvenot P, Vales G, Tessaud-Rita N, Maury MM, Alexandru A, Criscuolo A, Quevillon E, Donguy MP, Enouf V, de Valk H, Brisse S, Lecuit M. 2017. Real-Time Whole-Genome Sequencing for Surveillance of *Listeria monocytogenes*, France. Emerg Infect Dis 23:1462-1470.
4. Arndt D, Grant JR, Marcu A, Sajed T, Pon A, Liang Y, Wishart DS. 2016. PHASTER: a better, faster version of the PHAST phage search tool. Nucleic Acids Res 44:W16-21.
5. Leisner JJ, Larsen MH, Jorgensen RL, Brondsted L, Thomsen LE, Ingmer H. 2008. Chitin hydrolysis by *Listeria* spp., including *L. monocytogenes*. Appl Environ Microbiol 74:3823-30.
6. Chaudhuri S, Bruno JC, Alonzo F, 3rd, Xayarath B, Cianciotto NP, Freitag NE. 2010. Contribution of chitinases to *Listeria monocytogenes* pathogenesis. Appl Environ Microbiol 76:7302-5.
7. Ling Z, Zhao D, Xie X, Yao H, Wang Y, Kong S, Chen X, Pan Z, Jiao X, Yin Y. 2021. *inlF* Enhances *Listeria monocytogenes* Early-Stage Infection by Inhibiting the Inflammatory Response. Front Cell Infect Microbiol 11:748461.
8. Gaillard JL, Berche P, Frehel C, Gouin E, Cossart P. 1991. Entry of *L. monocytogenes* into cells is mediated by internalin, a repeat protein reminiscent of surface antigens from gram-positive cocci. Cell 65:1127-41.
9. Cabanes D, Dussurget O, Dehoux P, Cossart P. 2004. Auto, a surface associated autolysin of *Listeria monocytogenes* required for entry into eukaryotic cells and virulence. Mol Microbiol 51:1601-14.
10. Balogh D, Eckel K, Fetzer C, Sieber SA. 2022. *Listeria monocytogenes* utilizes the ClpP1/2 proteolytic machinery for fine-tuned substrate degradation at elevated temperatures. RSC Chem Biol 3:955-971.
11. Sanchez-Campillo M, Dramsi S, Gomez-Gomez JM, Michel E, Dehoux P, Cossart P, Baquero F, Perez-Diaz JC. 1995. Modulation of DNA topology by *flaR*, a new gene from *Listeria monocytogenes*. Mol Microbiol 18:801-11.
12. Heuston S, Begley M, Davey MS, Eberl M, Casey PG, Hill C, Gahan CGM. 2012. HmgR, a key enzyme in the mevalonate pathway for isoprenoid biosynthesis, is essential for growth of Listeria monocytogenes EGDe. Microbiology (Reading) 158:1684-1693.
13. Eugster MR, Haug MC, Huwiler SG, Loessner MJ. 2011. The cell wall binding domain of *Listeria* bacteriophage endolysin PlyP35 recognizes terminal GlcNAc residues in cell wall teichoic acid. Mol Microbiol 81:1419-32.
14. Rismondo J, Schulz LM, Yacoub M, Wadhawan A, Hoppert M, Dionne MS, Grundling A. 2021. EslB Is Required for Cell Wall Biosynthesis and Modification in *Listeria monocytogenes*. J Bacteriol 203.
